# The association between sleep deprivation and arterial pressure variations: a systematic literature review

**DOI:** 10.1016/j.sleepx.2022.100042

**Published:** 2022-01-26

**Authors:** Alécio Vinícius Sá Gomes e Farias, Mariana Peixoto de Lima Cavalcanti, Marcelo Alcântara de Passos Junior, Bruna del Vechio Koike

**Affiliations:** Universidade Federal Do Vale Do São Francisco (UNIVASF), Colegiado de Medicina (CMED), Petrolina (PE), Brazil

**Keywords:** Sleep privation, Hypertension, Blood pressure and arterial pressure

## Abstract

**Objectives:**

Arterial hypertension is a cardiovascular disease defined as a sustained high blood pressure, constituting an important risk factor for the development of heart diseases, such as coronary heart disease and heart failure. At the same time, pathophysiological pathways underlying sleeping deprivation provides biological plausibility for a causation connection between sleep deprivation and acute or chronic blood pressure elevation, such as the mechanism behind blood pressure dipping at night, which strongly relies on reduced sympathetic activity provided by sleep, besides empirical and clinical evidence suggesting that sleep disorders incidence is correlate with posterior development of arterial hypertension. The aim of this study was to systematically review published studies analyzing the possible relationship between sleep deprivation and variations in blood pressure during nighttime and daytime.

**Methods:**

The research was carried out in the second semester of 2020 following the PRISMA model and using the LILACS, MEDLINE and COCHRANE (CENTRAL) databases. The keywords used were associated using the Boolean method. Only trials and studies in humans unrelated to sleep apnea were included, in an attempt to answer the question proposed. Duplications and articles outside the topic were excluded.

**Results:**

After the selection processes, fourteen studies were left, which were classified, depending on the findings, in four categories: 1) blood pressure differences only in sleep deprivation's night; 2) blood pressure differences only in the following day after sleep deprivation's night; 3) blood pressure differences in both nights and 4) those that found no blood pressure differences.

**Conclusion:**

It was found an increase in blood pressure on the night of sleep deprivation, suggesting a possible causality with an acute increase in blood pressure depending on the population studied. In general, sleep deprivation is acutely associated with blood pressure elevation or acute elevation of markers that suggest the role of compensatory mechanisms, such as increased natriuresis and increased parasympathetic activity.

## Highlights

• Sleep deprivation results in increased simpathetic activity.
• Chronic sleep deprivation might lead to systemic arterial hypertension.
• Increased parasympathetic activity in acute sleep deprivation play a role as compensatory mechanism.

## 1 Introduction

The relationship between sleep disorders and blood pressure measurement is an important issue, mainly because it is a possible modifiable cardiovascular risk factor. Sleep apnea is already recognized as one of the most prevalent causes of secondary hypertension and, accordingly, deprivation has become a study target in recent decades [1]. Observational studies have already reported a significant association between the decrease in total hours of sleep and the emergence of hypertension [2], and thus, controlled studies list instruments to consolidate this association.

Arterial hypertension (AH) is a clinical condition characterized by a sustained blood pressure (BP) elevation, in which the treatment aiming to decrease the pressure levels provides greater benefits than risks, as indicated by clinical trials [3]. It constitutes an important risk factor for cardiovascular disease development, such as coronary heart disease and heart failure [3]. In most patients, it is not possible to identify a primary cause of the chronic elevated BP, usually called primary hypertension, known to be a multifactorial disease. In this scenario, both intrinsic, such as hormonal activation, and extrinsic factors, such as sleep habits, may play an important role in the pathophysiology of the disease [4]. The literature about the relationship between sleep disturbances and the subsequent occurrence of cardiovascular disease and overall mortality is vast [5,6].

Sleep deprivation (SD) might play an important role in acute or chronic blood pressure elevation, once known that SD is associated with relevant alterations in neuroendocrine function. This link is supported by the increased cortisol and lipids levels after several days of sleep deprivation, suggesting disturbance in the hypothalamic–pituitary–adrenal axis [7]. Furthermore, the duration of wake after sleep onset (WASO) is directly related to heart rate, blood pressure, cortisol in saliva, decreased 24 h leptin levels, total cholesterol, LDL-C and LDL/HDL-ratio [8]. These observations imply the possibility of a strong relation between chronic disturbed sleep and posterior diagnosis of metabolic syndrome, due to the development of AH, diabetes mellitus and dyslipidemia, suggesting metabolic and autonomic changes after sleep deprivation.

The causality between sleep apnea and AH has been widely described, especially due to sustained over sympathetic activation secondary to apneic moments during sleep, leading to a disturbed arterial levels of oxygen and carbon dioxide, resulting in lower baroreceptor sensitivity, changes in water-salt metabolism and vascular responsiveness [5,9]. Thus, official guidelines on hypertension and other cardiovascular diseases have established specific diagnostic protocols and treatment for patients presenting sleep apnea in association with other diseases.

An important variable is the possibility of compensatory mechanisms, such as concomitant vagal activation, to suppress an overall increased blood pressure in acute SD, even though there is an increased sympathetic response, with the possibility of a long term high blood pressure outcome after continued exposure to this condition [4].

The exact magnitude and mechanisms of the general association between sleep quality and AH have to be elucidated. As a consequence of current evidence, the primary aim of this study is to systematically review published studies analyzing the possible relationship between sleep deprivation and blood pressure variations during nighttime and daytime or hypertension itself.

## 2 Material and methods

### 2.1 Information sources and search strategies

A systematic literature search was undertaken following PRISMA methodology [10] from inception of the databases until 10/12/2020, in LILACS (Latin American and Caribbean Literature in Health Sciences), MEDLINE (International Literature in Health Sciences) and COCHRANE (Collection of Health Banks). The descriptors used were the Mesh Terms and DEC Terms for Sleep Deprivation, Hypertension and Arterial Pressure in English and Portuguese. The search was performed by associating the keywords with the Boolean operators “AND” between descriptors of different classes and “OR” between descriptors of the same classes, thus forming the search algorithm. Study and test filters were applied, covering all types, equivalent across platforms.

### 2.2 Eligibility criteria

Studies related to animal testing and sleep apnea were excluded, as were those that did not intend to address the relationship between sleep and BP. The studies were selected if they met the following criteria: being a controlled study or trial, searching the possible pathophysiological mechanisms resulting from SD and changes in BP, with the response in humans as the source of the study/trial and having an explicit protocol of SD. Duplicates, articles unrelated to the topic addressed and reviews were excluded.

### 2.3 Selection of studies and data extractions

Taking the eligibility and exclusion criteria, the studies were selected in two steps: first, reading the title and abstract and, second, reading the full text. Disagreements were discussed and defined by consensus among the authors.

Three authors extracted data independently from each study focusing on the first author's name, sleep deprivation protocol, sample and results. Obtained data was organized in tables disposed along with results.

### 2.4 Risk-of-bias assessment

The *Risk of bias for non-randomized studies of interventions* (ROBINS-I) [11] was used to assess the risk of bias of non-randomized studies. On the other hand, *Chocrane risk-of-bias toll of randomized trials* (RoB 2) [12] was used to assess studies presenting randomization. It was set that high-risk bias studies would not provide evidence strong enough to draw consistent conclusions. Two authors made the assessment independently; disagreements were debated and resolved by consensus among all authors. In general, studies were not blinded due to the intrinsic nature of the subject. Results are summarized in Table 1.

**Table 1:**
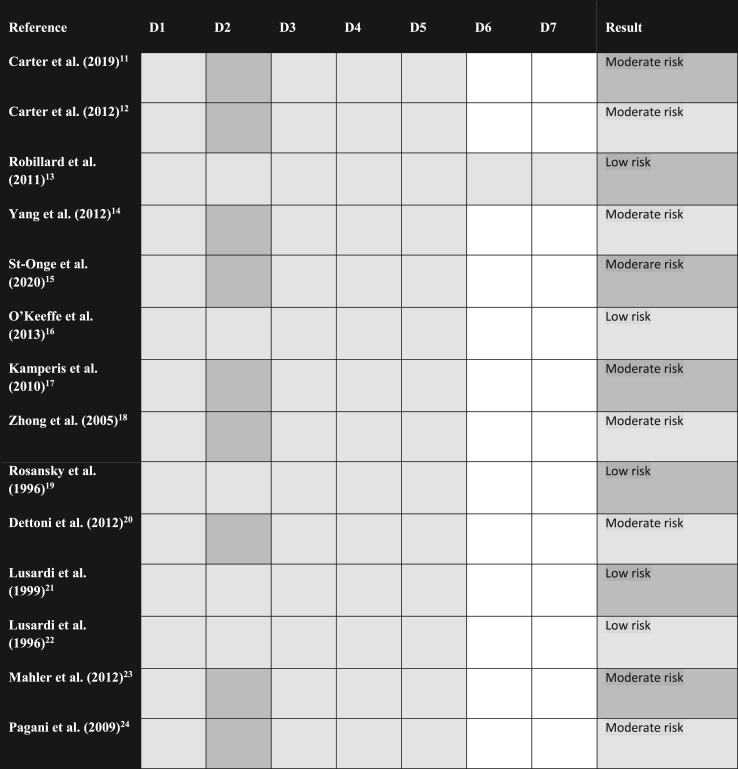
Risk-of-bias assessment.

Note - D1: Risk of bias for the RoB2 randomization process and risk of confounding bias for ROBINS I; D2: Risk of bias due to deviations from the intended intervention for RoB2 and risk of bias in participant selection for ROBINS I; D3: Risk of bias due to lack of outcome data for RoB2 and risk of bias in classification and interventions for ROBINS I; D4: Risk of bias in the outcome measures and risk of bias due to deviations from the intended intervention for ROBINS I; D5: Risk of bias in the selection of reported results and risk of bias due to missing data for ROBINS I; D6: Not applicable for Rob2 and risk of bias for outcome measurement for ROBINS I; D6: Not applicable for Rob2 and risk of bias when selecting results reported for ROBINS I. Light gray represents low risk of bias, dark gray represents moderate risk of bias, and dotted filling represents high risk of bias (there were no studies in this classification).

### 2.5 Analysis of the results

The prioritized information included in the summarized tables, was the ones that directly analyzes the relationship between SD and BP levels, including the biochemical and physiological parameters related to the pathophysiology of AH and its association with SD. Bias analysis was also important to stratify the quality of the evidence reported.

## 3 Results

**Fig. 1:**
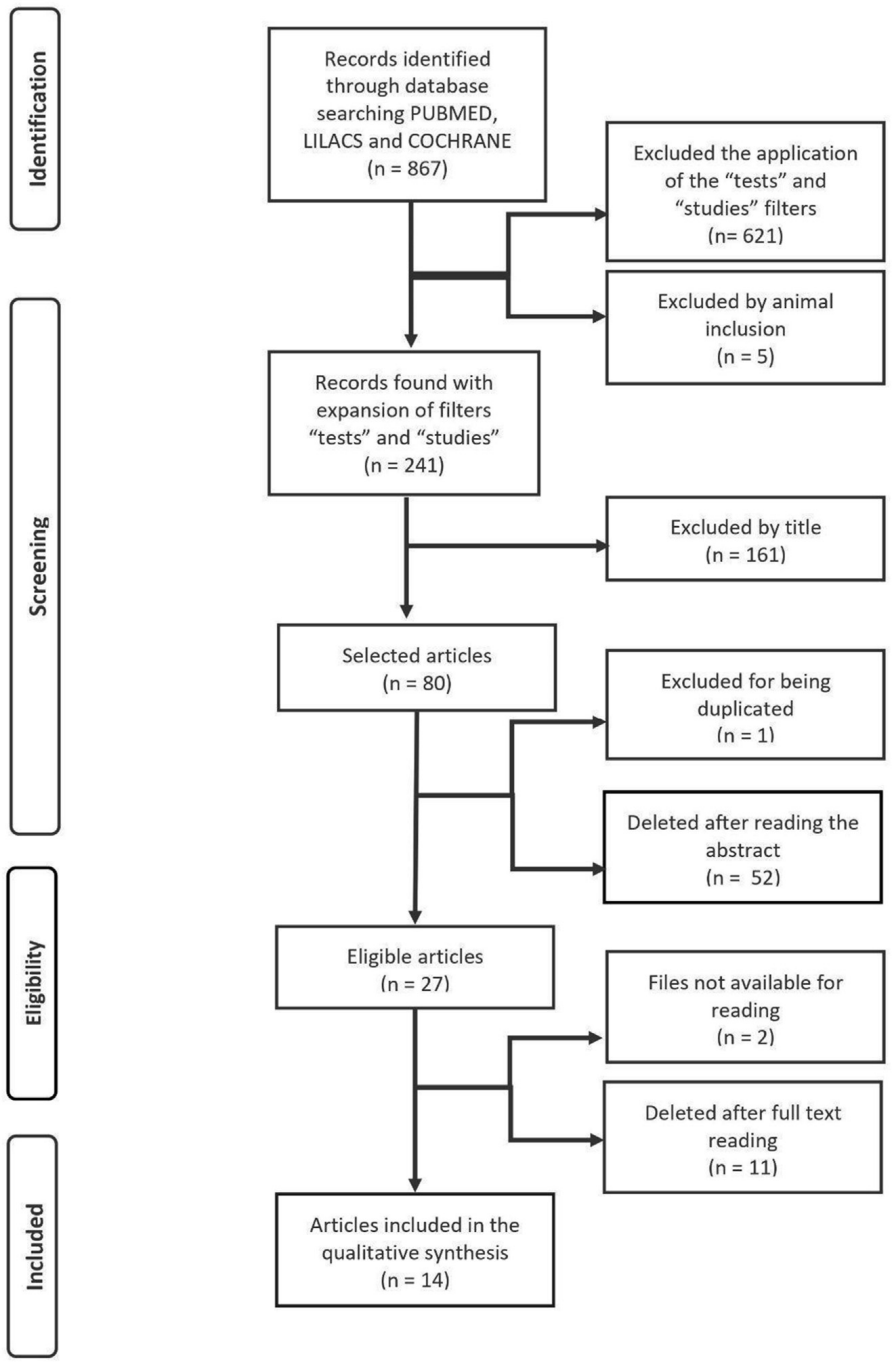
PRISMA flowchart representing literature's search and selection steps.

In total, this systematic review resulted in 867 papers (Fig. 1). In the handling of the different platforms tools, only non-reviews articles were filtered, resulting in 241 articles. 5 papers from those selected were excluded because they were performed on animals. In the exclusion processes, 161 were excluded after reading the titles, 1 was duplicated, 52 after reading the abstracts, 2 because the articles were not fully available and 11 after reading them in full. Finally, fourteen studies were left, which were summarized and could be classified in 4 groups, accordingly the results/findings: 1) that found BP differences only in the SD's night, 2) those that found BP differences only in the following day after SD's night, 3) those that found BP differences in both days and 4) those that found no BP differences.

### 3.1 Blood pressure differences only in sleep Deprivation's night

The BP dipping at night is a physiological phenomenon that results from reduced physical, mental and sympathetic activity, constituting a strong predictive value of cardiovascular risk and a well-documented relationship with sleep quality [13].

The lack of BP dipping has been associated with diseases that interfere with sleep patterns, such as sleep apnea, suggesting sympathoexcitatory mechanisms [14]. From this perspective, an increase in BP during SD is theoretically expected, especially due to the lack of sleep immersion. (see Table 2).

**Table 2:** Characteristics of selected studies for the outcome of Blood Pressure differences only in sleep deprivation's night.

Author	Sleep Deprivation Protocol	Sample	Results
Mahler et al. (2012) [15]	24 h sleep deprivation.	20 healthy volunteers. Aged 8–12 yr, Tanner pubertal stage 1 or 2, presenting a normal physical examination.	Daytime BP and heart rates were the same on both days. SD resulted in higher nighttime levels of blood pressure and heart rate.

BP: Blood pressure; SD: Sleep Deprivation.

In healthy children undergoing SD protocol, BP and heart rate (HR) present the same mean during daytime, on both days, even though the SD resulted in higher nighttime BP and HR level [15]. SD has been shown to play an important role in nocturnal diuresis, resulting in an increase of 68% in urinary volume on average (P < 0.001) [15]. A marked increase in clearance, total and fractional excretion of sodium, as well as decreased concentration levels of nocturnal plasma renin and angiotensin II were present in children going through SD, which may have played a compensatory role in maintaining regular BP in the following morning. It is also important to highlight that the authors suggested that BP levels returned to normal levels in the last hour of SD, probably because some children fell asleep, decreasing methodological study's strength. It is suggested that excessive nocturnal diuresis is possibly a consequence of higher BP levels in SD.

### 3.2 Blood pressure differences in the day after sleep Deprivation's night

Seven studies found blood pressure differences only the day after sleep deprivation's night, being included in this set those that measured BP in both moments and observed significant difference in the next day and those that measured BP only in the following day (Table 3).

**Table 3:** Characteristics of selected studies for the outcome of Blood Pressure differences in the day after Sleep Deprivation's night.

Author	Sleep Deprivation Protocol	Sample	Results
Carter et al. (2012) [16]	One night of sleep and one night of sleep deprivation separated by 1 month	28 individuals (age, 22 ± 1). (14 men and 14 women)	TSD induced similar increases in SAP, DAP and MAP in men and women
St-Onge et al. (2020) [17]	Randomized study comparing the usual sleep and reduced by 1.5 h a night for 6 weeks.	Healthy women between 20 and 79 years old	Higher scores assessed by the Berlin questionnaire were associated with higher PAD. SD increases SAP. No difference in SAP, DAP and mean arterial pressure during sleep and the physiological fall of SAP and DAP
Lusardi et al. (1996) [18]	Comparison between a normal night's sleep and a private night's sleep.	18 normotensive individuals (8 men and 10 women) between 24 and 30 years old.	From 2 am to 5 am, BP showed no differences between both conditions. At 6 am, BP significantly decreased, in discrepancy with non-private sleep. After waking up, PAS was significantly higher.
Kamperis et al. (2010) [19]	Randomized study with 2 phases (each with 24 h, and in one, sleep was not allowed).	20 healthy adults (10 women) aged 18–35 years.	TSD led to excess natriuresis (more pronounced in men than women) TSD reduced significantly activated renin and ANG II (similar in both sexes)
Yang et al. (2012) [20]	One group on Total Sleep Deprivation for 24 h s and the control group had a normal sleep. Randomized selection	28 healthy adults—14 men (age 22 ± 1 yr) and 14 women (22 ± 1 yr).	TSD increased the SAP baseline immediately before the mental stress and cold pressure test (CPT) assays and did not change HR at rest during the baselines before MS or CPT.
Carter et al. (2019) [21]	Randomized study in two phases: after 24 h of total sleep deprivation (TSD) and normal sleep (NS).	Twenty healthy elderly people aged 55–75 years (10 men and 10 women).	TSD increases blood pressure and SAP. TSD decreases HR TSD did not change DAP or MAP Significant sympatoexcitatory response in women, when quantified as an explosion rate, but not in men.
Robillard et al. (2011) [22]	One night of sleep and one night of sleep deprivation separated by > 2 weeks	16 normotensive individuals (8 between 20 and 28 years old and 8 between 60 and 69 years old)	SD increased SAP and DAP compared to healthy sleep in the elderly, but not in young people. SAP and DAP levels in elderly were those used in AH diagnosis.

TSD - Total Sleep Deprivation; SAP - Systolic Arterial Pressure; DAP - Dyastolic Arterial Pressure; MAP - Mean Arterial Pressure; CPT - Cold Pressure Teste; HR: Heart rate; SD: Sleep Deprivation; BP: Blood Pressure; ANG II: Angiotensin II; AH: arterial hypertension; MS: Mental stress.

In young individuals, TSD caused similar increases in Systolic Blood Pressure (SBP), Diastolic Blood Pressure (DBP) and Mean Blood Pressure (MBP) in men and women, regardless of body position, and BP dipping was largely attenuated, but it did not change the resting heart rate or spontaneous sympathetic baroreflex sensitivity [16]. Despite these findings in common between the groups, some variables were divergent or expressed differently between the sexes.

TSD caused a significant reduction in testosterone and progesterone, in young men, without altering estradiol levels. There was a decrease in Muscle Sympathetic Nerve Activity (MSNA), correlated with changes in testosterone [16]. The TSD also significantly decreased the point of operation in men, moving it down and to the right of the spontaneous sympathetic baroreflex operating point, which could not be seen in women. On the other hand, there was a more drastic reduction in progesterone, but not in testosterone or estradiol levels in female subjects [16]. Total SD did not significantly change the cardiovascular and sympathetic Baroreflex Sensitivity (BRS), nor the diastolic DBP and MBP. At the same time, post hoc analysis revealed that, in women, TSD significantly increased the frequency of bursts, that is, an increase in sympathetic excitement, frequency and incidence of MSNA outbreaks, which does not occur in men [21]. Such findings may help to explain why the relationships between SD and AH are stronger in women, suggesting that the mechanisms underlying the hypertensive response aggravate the TSD differently in men and women.

Psychological and hemodynamic aspects result in increased levels of wakefulness SBP and DBP and MBP in SD conditions, but without significant differences in these parameters during sleep. Thus, the results point to an inverse correlation between total sleep time and BP measurements during wakefulness [17]. On psychological stress evaluation, even though total sleep deprivation reveals increased SAP immediately before mental stress testing (P < 0,005), no significant differences in BP reactivity was observed [20].

Moreover, in elderly patients, SD did not significantly alter the levels of estradiol, progesterone and testosterone in both sexes [21], but there was an acute increase in BP and SBP in these individuals, reaching prehypertensive and hypertensive levels [22].

### 3.3 Blood pressure differences in both conditions

One of the selected studies could find significant increases in BP components during deprivation and the following day (Table 4).

**Table 4:** Characteristics of selected studies for the outcome of Blood Pressure differences in both days.

Author	Sleep Deprivation Protocol	Sample	Results
Lusardi et al. (1999) [23]	Normal sleep night (11 pm–7 am) vs SD night (3am–7am)	39 patients (20 men and 19 women) aged between 34 and 68 years, mild to moderate hypertension.	BP was higher during the day after SD BP was higher during SD and even higher in the morning after SD. Urinary norepinephrine excretion increased during SD.

BP: Blood Pressure; SD: Sleep deprivation.

Higher values for 24-h MBP during the day after sleep deprivation and BP during sleep deprivation and in the morning after sleep deprivation were observed, concomitantly there was also a reduction in BP dipping at night and increased state-anxiety in both man and woman. Increased norepinephrine during sleep deprivation suggests increased sympathetic activity, contributing to higher BP, even in partial SD on the first part of nightime [23].

### 3.4 No blood pressure differences found

Five studies have not shown differences in BP parameters before, during or after SD. In this set were included the articles that measured the variables in both moments and found no differences at all and also the articles that measured only in SD's night or the following day, without any information about the volunteer during the interval (Table 5).

**Table 5:** Characteristics of selected studies for the outcome of no Blood Pressure differences.

Author	Sleep Deprivation Protocol	Sample	Results
Pagani et al. (2009) [24]	Comparison between 8 days in a monotonous work environment and sleep deprivation in low light conditions, performing a simple computerized task every 2 h.	24 young healthy subjects (12 male, 12 female; age 27–45)	After one night of SD, there are no signs of increases in BP.
O'Keeffe et al. (2013) [25]	Habitual sleep (22:00–07:00 h s) vs SD (01:00–05:00 h s)	14 men and 13 women, aged 30–45 y, were recruited via approved media.	Effects of sleep on systolic and diastolic blood pressure and RHR were not significant.
Dettoni et al. (2012) [26]	5 nights of control and 5 nights of SD	13 healthy male volunteers between 21 and 45 years old (age: 31 ± 2 yr)	SD did not significantly change HR, BDP and DBP.
Rosansky et al. (1996) [27]	Patients were monitored after sleep deprivation (<4 h of sleep) and after a normal night's sleep (at least 5 ½ hours of sleep).	24 normotensive individuals with an average age of 29.3 years.	There was no difference in mean day, night and 24-h BP.
Zhong et al. (2005) [28]	The sleep records kept for 2 weeks before the laboratory phase of the study were compared with the TSD phase (36 h of sleep deprivation). Subjects were kept awake by verbal stimuli	Eighteen subjects (2 women, 16 men), aged 26.0 ± 4.6 years, were recruited by advertisements.	No significant change in BP baseline Significant decline in HR Significant decline in RR Increase in normalized LF

SD - Sleep Deprivation; HR - Heart Rate; SAP - Systolic Arterial Pressure; DAP - Diastolic Arterial Pressure; BP: Blood pressure; RR: respiratory rate LF: low-frequency; RHR: Resting heart rate.

Comparing the same group of subjects in two different conditions, in which one has a monotonous ambient work with a complete SD night and other has a normal life routine at home, with a strict sleep-wake schedule; they aimed to analyze the hemodynamic aspects, autonomic variations and baroreflex control. The results reveal no signs of increase in arterial pressure variability, nonetheless it was suggested an increase in cardiac vagal regulation, which may possibly act as a compensatory mechanism against BP raise [24]. In the same direction, insignificant effects were found in a two phase randomized study which compared habitual sleep and short sleep [25,26], concomitant to the lack of differences in mean day, night and 24-h BP, founded by other researchers [27].

Even though no BP significant changes in baseline was observed between SD and non-SD groups, there was an increase in sympathetic cardiac modulation and BP, less parasympathetic modulation and less spontaneous baroreflex sensitivity (BRS) in 12-h of nocturnal SD [28].

In summary, it seems that healthy adults going through one night SD protocol, but in the absence of significant stress, might not present significant BP increase.

## 4 Discussion

Observational studies of sleep deprivation have demonstrated graded associations between higher SBP and DBP and increased Cardiovascular Disease (CVD) risk [29,30]. The risk of CVD increased in a log-linear fashion from SBP less than 115 mmHg or more than 180 mmHg and DBP levels less than 75 mmHg or more than 105 mmHg [31].

A set of articles found arterial pressure alterations only during the TSD night. A possible relationship between high BP and TSD is observed, while compensatory mechanisms are detected acting in the bypass way, such as increased natriuresis and decreased levels of angiotensin II and renin [20]. These mechanisms may play a role in maintaining normal BP levels in the morning, standing out as possible justification for the absence of changes in this period [31]. An association between acute SD and an increase in sympathetic cardiac modulation and BP levels have been suggested, as well as a parasympathetic activation reduction and a consecutive decrease in the baroreflex sensitivity [28].

Meanwhile, one article reported significant increases in BP variables during TSD and in the following day. Although it does not propose a specific mechanism, it suggests an increase of the rapid eye movement (REM) sleep duration, in which the increased sympathetic activity resulting from sleep rebound contributes to the significant increase in mean BP [32]. In addition, the TSD protocol can be characterized as a stressful condition [33], which, at first, tends to increase the values of serum catecholamines [20], a hypothesis sustained, mainly, by the increase in urinary norepinephrine excretion [20].

On the differences between sexes in influencing their levels of BP, there is a possible correlation in MSNA response to TSD, as well as the DBP-MSNA relationship [21]. In young men, the sympathetic inhibition generated by the TSD is linked to the mechanism responsible for the increase in BP, reflecting a possible reset of the sympathetic baroreflex, this mechanism being associated with the reduction in testosterone levels that occurred in response to the TSD [16]. In contrast, the mechanism responsible for the arterial pressure increase in women, in which there is no decrease in MSNA, would be linked to some degree of baroreflex dysfunction [16].

When compared to men of the same age, women tend to exhibit lower baseline MSNA in early adulthood (20–39 years), more or less similar levels of mid-life MSMA (40–49 years), and significantly elevated levels of MSNA at rest in adulthood (>60 years) [10,11]. In addition, it was possible to identify increases of up to three times in MSNA every decade of a woman's life when compared to men, due to the increase in sympathetic tone, thus contributing to BP dysregulation [21].There's a clear correlation between MSNA and age influenced by sex [34] “Cardioprotection” would occur in young women as result of an α-adrenergic vasoconstriction performance offset by β-adrenergic vasodilation in early life before an aggressive increase in cardiovascular risk during the post-menopausal years [35]. As result, conditions that cause increased MSNA in older adults, and particularly in women, are clinically relevant to the change in cardiovascular risk.

Considerations about estradiol, are based on the incidence of hypertension in postmenopausal women is four times higher than premenopausal [36], probably due to reductions in estradiol production [37]. This is attributed to the fact observed in the variations of estradiol during the menstrual cycle associated with BP. Further, it is reported that the replacement of this hormone has BP-lowering effects [38], although some recent studies contradict this statement [39].

A possible correlated biomarker would be an increase in the concentration of norepinephrine in a positive MSNA relationship, as suggested by Wallin et al. [40], which can be reinforced by the findings of Carter et al. [21] of excitatory hyperactivity of MSNA in response to SD.

In those studies that did not show any difference in BP during and after SD, some compensatory mechanisms that could explain these phenomena have been proposed by some authors, such as increased cardiac vagal activation [23,41] and venous endothelial dysfunction [42,43]. One [27] of these studies was not able to detect the compensatory physiological decline of BP at night after SD, which can interfere in the clinical development of AH if the subject is exposed to recurrent deprivation.

Differences in sleep deprivation protocols can be identified as one of the main determinants of variations in the results shown in this systematic review. The study by Mahler et al. [15] was originally designed for the assessment of diuresis and natriuresis in healthy children in a condition of sleep deprivation. Thus, the patients were in a supine position with prohibited physical activities, food and liquids. Probably, the findings of this study are due to the specific design, which induced suppressed plasma levels of sodium-retaining hormones, a mechanism already associated with blood pressure control [31]. Blood pressure measurements in the two studies by Carter et al. [16,21] and in the articles by Yang et al. [20] and Robillard et al. [22] were only done in the days following sleep deprivation, thus, there are no nocturnal records of these studies. With a similar design, St-Onge et al. [17] performed weekly and only daytime measurements. Such study conditions did not consider the nocturnal pressure characteristics, conditioning a result with only daytime data. The study by Kamperis [19] used the ambulatory blood pressure monitor, then monitored blood pressure for 24 h, also adding the period in question, however, without finding significant changes. Lusardi et al. using a similar protocol, reached this result in 1996 [18], and in 1999 [23], with a larger number of participants and an increase in the study exclusion criteria (secondary hypertension, sleep apnea syndrome, organic damage and use of antihypertensive agents or cardiovascular drugs), found significant pressure changes also on the deprivation night. Studies that did not demonstrate significant changes in blood pressure in any condition were the majority and had a greater number of total participants. Three of the five studies lasted for 6 or more days [[24], [25], [26]] and the other two standardized the participants' diet [25,28]. Such characteristics, added to the number of patients, even though only Zhong et al. [28] and Rosansky et al. [27] use 24-h monitoring, establish greater control of the study and, theoretically, explain results that are more in line with the reality. However, the differences between the previous results is expected and common in articles of this type. Variations in study designs and a strong randomness factor, potentiated by the low number of individuals subjected to the conditions described, make it difficult to analyze the few studies in the area, reinforcing the particular need to assess sleep architecture and its various presentations in different environments and clinical conditions.

There are several mechanisms responsible for BP maintenance, neural in nature, linked to moment-to-moment BP control, and humoral, related to long-term BP control [44]. The first is performed mainly through control areas in the brainstem (vasomotor center) and the efference of the autonomic nervous system (sympathetic and parasympathetic), acting on the vessels and the heart, where it acts in the modulation of cardiac output (ventricular filling, inotropism and chronotropism) [44]. The main reflexes involved in this pressure control are the baroreflex, the cardiopulmonary reflexes, the chemoreflexes and the renorenal reflex, whereas with regard to the humoral control, the main mechanisms involve the renin-angiotensin system [44].

These regulatory mechanisms are affected by the state of sleep and wakefulness, in which the vasomotor center acts in sympathetic and parasympathetic modulation, through the modification of peripheral vascular resistance, venous return, heart rate, contractility and cardiac output [45]. In this context, the alterations observed during sleep are due to the functional integrity of the baroreflex, which suffers a drop in its sensitivity threshold in this state, especially during the NREM (non-rapid eye movement) phase [46]. It is uncertain whether this change during sleep is due to an induction or passive consequence of physical and cognitive inactivity during sleep.

Therefore, SD interferes with the regulatory mechanisms of BP, either because it interferes with the baroreflex mechanism and does not allow the BP to fall at night [16,21] or because of the increase in MSNA, acting also on the renin-angiotensin system that acts in a compensatory attempt to increase pressure levels due to SD [19].

Evidence on the association between sleep deprivation and higher blood pressure levels converge in the point that, regardless of age or sex, on avarage all individuals tends to present oversimpathetic activation, presenting increased cortisol, systemic cathecolamines and sodium regulating hormones, even though acute blood pressure variations respond diferently among diferent individuals and diferent SD protocols. BP variations according to age seem to be related to the ongoing decrease in compensatory capacity for overcoming sympathetic drive secondary to sleep deprivation.

It is also important to highlight that this systematic review presents some limitations: 1) Although they have been exhaustively searched for, some full texts could not be found in databases used, which may have eliminated some articles that could have been included and used in qualitative synthesis. 2) Some studies have methodological flaws, introducing risks of biases in these trials, at the same time that some did not have physiological parameters, such as blood pressure levels, measured during and the day after sleep deprivation, concurrently. This strategy may have ignored important data, since the situations were not compared, thus some conclusions could not be clearly made. 3) Only papers in English were collected, which possibly narrowed our results and more databases were available to search. 4) We only included studies that describe objectively the sleep deprivation protocol. This allowed us to collect more comparable data among the articles, however, there are multiple studies that measure different kinds of sleep disturbances, such as microarousals, and its influence in blood pressure, presenting interesting results.

## 5 Conclusion

**Fig. 2:**
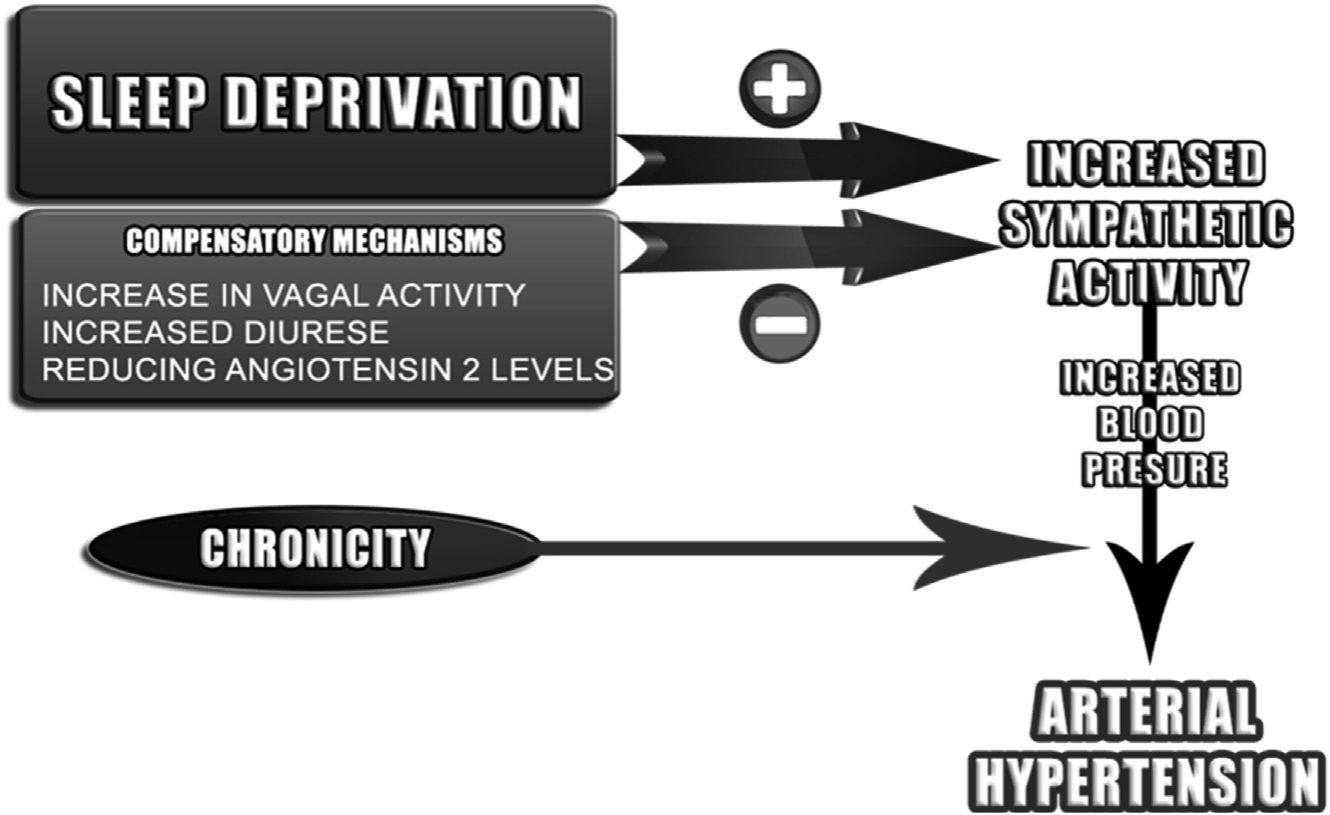
Schematic representation of chronic sleep deprivation together with compensatory mechanisms that may lead to arterial hypertension.

Given the proposals made, it is possible that acute SD can cause acute changes in BP or, at least, activate physiological compensatory pathways that could increase it. Several mechanisms have been described for such alterations and, even when the alterations were not detectable, the causality of the physiological compensation could be attributed or measured. It can then be hypothesized that repeated SD, in long term and in certain social contexts, could contribute to a persistent increase in sympathetic activation, even overcoming the autonomic parasympathetic mechanisms, which could lead to possible metabolic and cardiovascular outcomes such as AH and contribute for others, as suggested in Fig. 2. However, more studies on the subject are nedded to investigate the hypothesis of this correlation and to quantitatively establish the causality, until then hypothetical, and thus may base new therapeutic and preventive approaches if it is sustained.

## Funding sources

This research did not receive any specific grant from funding agencies in the public, commercial, or not-for-profit sectors.
